# The Effects of Structured Physical Activity Program on Social Interaction and Communication for Children with Autism

**DOI:** 10.1155/2018/1825046

**Published:** 2018-01-15

**Authors:** Mengxian Zhao, Shihui Chen

**Affiliations:** ^1^School of Sports Media and Information Technology, Shandong Sports University, Shandong, China; ^2^Department of Kinesiology, Texas A&M University, Texarkana, TX, USA

## Abstract

The purpose of this study was to investigate the effects of structured physical activity program on social interaction and communication of children with autism spectrum disorder (ASD). Fifty children with ASD from a special school were randomly divided into experimental and control groups. 25 children with ASD were placed in the experimental group, and the other 25 children as the control group participated in regular physical activity. A total of forty-one participants completed the study. A 12-week structured physical activity program was implemented with a total of 24 exercise sessions targeting social interaction and communication of children with ASD, and a quasi-experimental design was used for this study. Data were collected using quantitative and qualitative instruments. SSIS and ABLLS-R results showed that an overall improvement in social skills and social interaction for the experimental group across interim and posttests, *F* = 8.425, *p* = 0.001 (*p* < 0.005), and significant improvements appeared in communication, cooperation, social interaction, and self-control subdomains (*p* < 0.005). Conversely, no statistically significant differences were found in the control group (*p* > 0.005). The study concluded that the special structured physical activity program positively influenced social interaction and communication skills of children with ASD, especially in social skills, communication, prompt response, and frequency of expression.

## 1. Introduction

The population of autism spectrum disorder (ASD) is growing dramatically worldwide today. People with ASD have deficits in social interaction, communication skills, repetitive, restricted, and stereotyped patterns of behaviors, and interests of activities [1], all of which have a major influence on children's development and their families. The prevalence of ASD in 1980 was approximately 1 in 10,000, and the number of people currently diagnosed with ASD has already reached more than tens of millions [2]. In 2014, the newest estimation of children with ASD in US was 1 in 68, nearly a 120% increase from 1 in 150 in 2000. The problem in China is also serious; 1.61% of children under the age of 15 years were reportedly affected by varying degrees of ASD [3], and, more important, there is lack of attention and awareness on individuals with ASD, even though many people are beginning to focus on issues of children with ASD in China. At present, although cure for ASD is still unavailable, a number of treatments and interventions can help children with ASD improve their social and communication skills and function better.

Physical activity plays a vital role in influencing people's life from many aspects, and this is especially important for children because it can help them improve not only their physical condition, but also their self-esteem, social skill, and behavior [4, 5] and establish a positive lifestyle for their future lifespan. Many studies have reported on the importance and benefits of exercise and physical activity and the risk of physical inactivity for children, especially for children with disabilities [6–8]. Children with ASD are perhaps in more risking situation because of their limited opportunities to participate in physical activities. These children have fewer opportunities to engage in physical activity and play with their peers because of their impairments in social interactions and communication skills [9, 10]. Children with ASD also tend to be used to passive type of activities (e.g., isolated play, parallel play, and orientation), and they are typically less active compared with their peers during their free time. Undergoing the typical children development stage, fewer opportunities to engage in physical activity are more likely to influence their behaviors [11].

Physical activity is an important contributor for health in populations with developmental disabilities, and the benefits of physical activities for children with ASD have been studied [12]. It has been found that physical activities help improve a number of the many deficits and challenges that children with ASD must confront [13, 14]. The study also indicated that participation in physical activity allows children with ASD to experience a fun activity with their peers and to develop critical interpersonal skills [15]. In addition, some results of studies reported that several benefits derived from physical activity are related to mental and psychosocial wellbeing; for instance, physical activity could improve psychological wellbeing [16, 17], leading to positive self-esteem, behavior, and happiness in children with ASD [4]. Moreover, evidence has also shown that physical activity has a direct influence on self-determination [18] and strength and to have specific positive effects on children's cognitive and adaptive abilities (e.g., on-task behavior and academic performance).

At present, researchers and experts believe that certain social activity oriented interventions and treatments can help children with ASD change their behaviors and function better in their normal environment. With the increasing awareness of ASD in many countries, a number of intervention programs have been devised, implemented, and found to be effective on certain typical behaviors of children with ASD (e.g., auditory training intervention, vitamin therapy, medication intervention, and music intervention) (Centers for Disease Control and Prevention, 2014). The standardized interventions and treatments targeting the behaviors of children with ASD have already yielded certain beneficial effects and achieved a moderate degree of success.

As the social activity related interventions involve many physical interactions, more and more researchers in many countries have also begun to focus on the effects of physical activity and exercise on fading and redirecting the autistic behaviors of children with ASD. Two programs use a land-based aerobic exercise program and an aquatic exercise program as an intervention to analyze the effectiveness of changing certain inappropriate behaviors of children with ASD. Positive effects of physical activity interventions have been found effective on social behavior and social function [19], communication enhancement [20], exercise ability, body mass index [12], sensory and feeling [19], stereotypical behavioral patterns [21], and physical exercise participation [22].

As the impairments and difficulties in social interaction and communication are the primary characteristics for children with ASD, a lack of social skill and communication skill can lead them to a social isolation or withdrawal situations [23]. Therefore, an intervention focusing on their social and communication skills is critical to the successful development of social, emotional, and improvement of communication skill of children with ASD. At present, little evidence is available about the utilization of modified games and physical activities as an intervention on fading and redirecting the autistic behaviors of children with ASD; few studies had been conducted especially in China. Therefore, this study was to investigate the effectiveness of a structured physical activity program on social interaction and communication skill of children with ASD.

## 2. Materials and Methods

### 2.1. Participants

Fifty children with ASD, age from five to eight years old, in a special school of Shandong province, were recruited based on the DSM-V criteria [1] and randomly divided into experimental and control groups. Twenty-five children with ASD were placed in the experimental group in which the structured physical activity program was used as intervention. The other twenty-five children with ASD as the control group participated in regular physical activity. Four children with ASD withdrew from the experimental group, and five children withdrew from the control group for different reasons. A total of forty-one participants (*N* = 41) completed the study. A comparison was conducted regarding the baseline data between the experimental group and the control group. The demographic variables between the experimental group and control group were listed in Table 1 (all *p* > 0.005).

### 2.2. Instruments

There were four instruments that had been used to measure the effects of structured physical activity program on social interaction and communication skills of children with ASD. These instruments included the following: The Assessment of Basic Language and Learning Skills- Revised (ABLLS-R) which consists of a comprehensive hierarchy of skills in 25 different and separated areas. For the current study, the area of social interaction with total 34 items was used to evaluate the social interaction of children with ASD. The Social Skills Improvement System Rating Scales (SSIS-RS) was used for evaluating social skills, problem behaviors, and the academic competence of students from the perspectives of teachers, parents, and students and for the improvement of social skills in child with social and behavioral problems [24]. The Parent Semistructured Interview Guide (PSSIG) and Volunteer Open-ended Questionnaires (VOEQ) were also used to collect the comments and feedback from parents and volunteers regarding the effects of the structured physical activity program, future directions, and recommendations.

### 2.3. Research Design and Procedures

**Figure 1:**
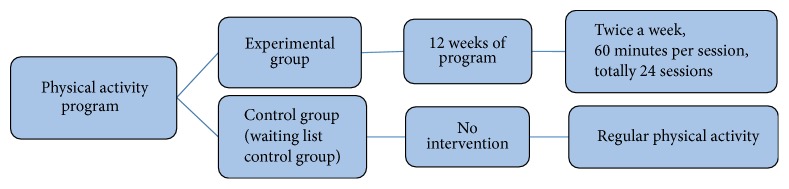
Framework of intervention.

A quasi-experimental design with a control group and an experimental group was utilized to investigate the effects of the 12-week structured physical activity program on social interaction and communication for children with ASD. The present study adopted the pretest, interim-test, and posttest design and conducted testing at three time intervals to monitor progressive changes in social interaction and communication throughout the 12-week intervention. The 12-week structured physical activity program was implemented with 60 minutes per session and total 24 exercise sessions. The experimental and control groups completed the pretest 1 week before the intervention program began. The interim-test was conducted halfway through the intervention, and the posttest was administered one week after the 12-week intervention program.  Figure 1 is the framework of intervention.

### 2.4. Intervention Program

This program has distinctive features and advantages. First, the structured physical activity program was designed based on guidelines and recommendations for children with ASD (e.g., DSM-V; treatments on autism; and the benefits of physical activity), specific recommendations on physical activity programs and children with ASD (e.g., Global recommendations on physical activity for health; current perspectives on PA, and exercise recommendations for children with ASD). Second, it was a structured program specialized in the needs of social interaction and communication, which comprised four parts: (a) get-ready and warm-up activities; (b) one-to-five small group instruction; (c) whole-group exercise; and (d) cool-down and reward activities.

In addition, all of the regular activities in this program were highly targeted towards the problems that were the focus of this study, and a number of useful and meaningful elements were purposely integrated into the program to create more opportunities to influence children's autistic behaviors. Moreover, all of the procedures and contents promoted the targeted behaviors. In brief, during the completion of each trial, each participant with ASD was “forced” to respond to peers greeting (e.g., peers' high-five, catching/passing ball from/to peers) and naturally communicate with peers (physical, verbal, or eye contact). When participants completed the entire session, they have experienced significantly increased frequency of their social interactions and communications with others. During each session, children with ASD were placed into a group of five, each group was assigned the same teacher, and each session was conducted using the same content and routine to provide the participants with a sense of consistency. The structured physical activity program was conducted in the fitness room or on the playground of the Special School of Shandong Province, and the school is equipped with many classrooms for various teaching purposes. The detailed contents of the structured physical activity program are listed in Table 2.

The intervention program also utilized the Treatment and Education of Autistic and Communication Handicapped Children (TEACCH) model to enhance its effectiveness (Panerai, Ferrante & Zingale, 2002). This model provided the individual with a structured and organized physical environment, the schedules and work systems, clear expectations, and the use of visual materials. Structured teaching also involved utilizing various strategies and principles for intervention and treatment.

The intervention program also included a reward system to encourage the children to be active and willing to participate in the physical activity program. For example, when the participants completed the exercise, they would be awarded with some tangible items; they are required/encouraged to say “Thank you” or “Bye,” after receiving the gift (communication and interaction). A part of each session was dedicated to the whole-group exercise; when they finished an exercise or won, the teacher gave them a high five. If they reacted by returning the high five, this was also considered an interaction. When the participants finished one part or the entire session, they received one sticker; once they collected a specific number of stickers, they could trade them in for a gift or an item they desired from the reward desk (e.g., toys and books); gifts were given according to the level they completed. Many purposely designed interactions and communications had been integrated into the physical activities intervention to enhance children's social/communication skills.

### 2.5. Data Collection and Analysis

In this study, the data, investigator, and methodological triangulation were applied. Data triangulation involved collecting data on the perspectives of the teachers, parents, and volunteers at different time points. Investigator triangulation was conducted by having more than one researcher analyze the data and having member checking. Methodological triangulation concerned the use of both qualitative and quantitative methods. The quantitative method was the primary method used, whereas the qualitative methods were supplementary, employed to demonstrate the effects of the structured physical activity program on social interaction and communication in children with ASD.

#### 2.5.1. Quantitative Data Collection and Analysis

Data collection was composed of four parts, as shown in Table 3. The first part involved the teachers from the special school, who conducted the pretest, interim-test, and posttest by administering the ABLLS-R [25] and SSIS-RS (Gresham & Elliott, 2008) at the scheduled time intervals. The quantitative scales were used to explore the participants' progress and the changes throughout the program, and the qualitative approach was used to supplement the findings.

The SSIS-RS and ABLLS-R data were analyzed through repeated measures MANOVA and two-way repeated measures ANOVA at three time intervals to determine the differences between the experimental group and the control group regarding social interaction and communication in the SSIS-RS and ABLLS-R.

Means and standard deviations at each time within each domain and subdomains were calculated. The primary analysis was to identify the difference between control group and experimental group after 12 weeks of physical activity program intervention.

#### 2.5.2. Qualitative Data Collection and Analysis

The semistructured interviews and open-ended questionnaires were distributed to the parents and volunteers, respectively, after the 12-week physical activity program. To ensure in-depth results and findings that were persuasive as well, the qualitative methods of in-depth semistructured interviews including external reviewers and member checking were performed for a more effective examination of the children's progress throughout the program.

Once the interviews were transcribed, member checking was conducted to present the participants with an opportunity to add, clarify, or rephrase the questionnaire responses (Lincoln & Guba, 1985). We added questions to the margins wherever further clarification was required. Each participant was free to decide whether he or she wanted to address the researcher's comments. The parents and volunteers were also asked to return the member checking data directly to the researcher within 1 week of having received the transcript.

The primary objective of this study was to determine the effects of the physical activity program on social interactions and communication in children with ASD. After data collection, SPSS (version 20.0) was used for data analysis, and it was used to evaluate the three sets of testing data, obtained by using the SSIS-RS and ABLLS-R instruments. The qualitative approach involving the semistructured interviews and open-ended questionnaire was analyzed using the NVivo (version 8) qualitative software package.

## 3. Results

### 3.1. The Effects of Structured Physical Activity Program on Social Interaction

#### 3.1.1. Effects of Intervention on SSIS over Time

The Social Skills Improvement System Rating Scales was adopted to evaluate the social skill improvement of children with ASD, and the repeated MANOVA was used to examine the effects of intervention on social skills and subdomains in SSIS. The results of repeated MANOVA showed that there were significant differences on the time effect, Wilk's Lambda = .156, *F*(2,38) = 102.461, (*p* < 0.05), as well as time × group interaction effect, Wilk's Lambda = .342, *F*(2,38) = 36.52, (*p* < 0.05). The data indicated that the social skills of children with ASD in experimental group were significantly improved after 12-week intervention.

#### 3.1.2. The Comparison of Effects of Intervention between-Groups on SSIS across Three Tests

*(1) Overall Social Skills*. In relation to the social skill scores in the SSIS between the experimental group and control group, a two-way repeated measures ANOVA was conducted to examine whether the structured physical activity program resulted in an increase in social skills between the groups across pretest, interim-test, and posttest (see Table 4).

Before the intervention, no statistically significant differences were found between the experimental and control groups in overall social skills. As shown in Table 4, in the interim-test, although there some changes occurred, there were no significant differences between the groups (*p* > 0.005).

After the 12-week physical activity program, a significant improvement was found in social skill scores for the experimental group compared with the control group (*p* < 0.005), which had a statistically significant effect. These results indicated that the structured physical activity program had a positive influence on social skills of children with ASD and improved their overall social skill scores in the SSIS.

**Figure 2:**
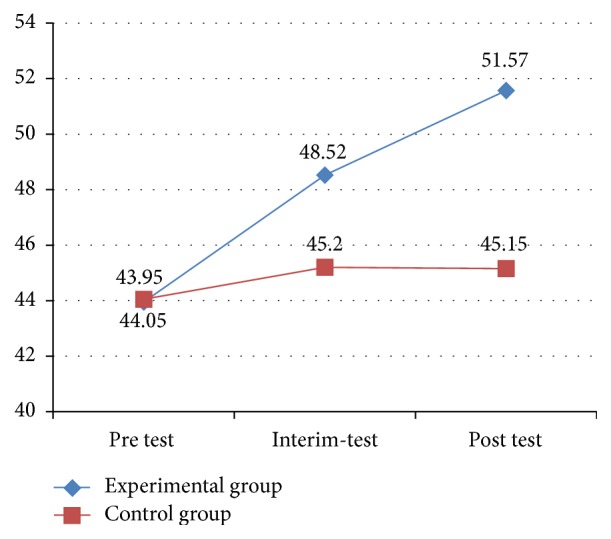
Results of social skills in SSIS between two groups.

Figure 2 displays the improvement trends in social skill scores for the experimental and control groups. The figure shows that the experimental group exhibited a more noticeable improvement over time, with significant differences in social skills from the pretest to the interim-test, and then to the posttest. By comparison, no significant differences were found in social skills among three testing periods for the control group.

*(2) Subdomains of Social Skills*. The social skill in the SSIS contains 46 items and it is categorized into seven subdomains: communication, cooperation, assertion, responsibility, empathy, engagement, and self-control.

Follow-up ANOVAs were conducted to measure whether the structured physical activity program led to an increase in the seven social skill subdomains in the SSIS between the groups across three intervals.

As shown in Table 5, there was a significant increase in communication, cooperation, and self-control from pretest to interim-test, and then to posttest compared with the control group (*p* < 0.005). These results revealed that after 12 weeks intervention, the structured physical activity program significantly enhanced the scores in the communication, cooperation and self-control subdomains of social skills in the SSIS.

#### 3.1.3. Comparison of Effects of Intervention within-Group on Overall Social Skills across Three Tests

The scores of overall social skills for experimental and control groups were measured across three various time points including pretest (T1, one week before the intervention), interim-test (T2, week 7), and posttest (T3, one week after the intervention).

As shown in Table 6, two-way repeated measures ANOVA was conducted to determine whether a significant difference existed at the three time points for the within-group comparison. The results revealed significant differences from T1 to T3 for the experimental group (*p* < 0.005). Furthermore, the overall social skill scores observed at T3 were significantly higher than those at T1 (*p* < 0.005). By comparison, the control group exhibited no statistically significant differences in the overall social skill scores across the three time points (all *p* < 0.005). These results revealed that the structured physical activity program had a positive influence on social skills in the experimental group and improved their overall social skill scores in the SSIS.

### 3.2. Effects of Intervention on ABLLS-R across Three Test Intervals

#### 3.2.1. Between-Group Comparison of Overall Social Interaction across Three Test Intervals

In relation to the social interaction scores in ABLLS-R between the experimental group and control group, two-way repeated measure ANOVA was conducted to examine whether the structured physical activity program resulted in an improvement in social interaction between the two groups. As shown in Table 7, the mean scores of social interaction were similar between two groups at baseline; the mean scores and standard deviations were 24.15 ± 3.34 and 23.81 ± 3.43, respectively. In the interim-test and posttest, the experimental group obtained significantly higher scores on social interaction in ABLLS-R (*p* < 0.005).

**Figure 3:**
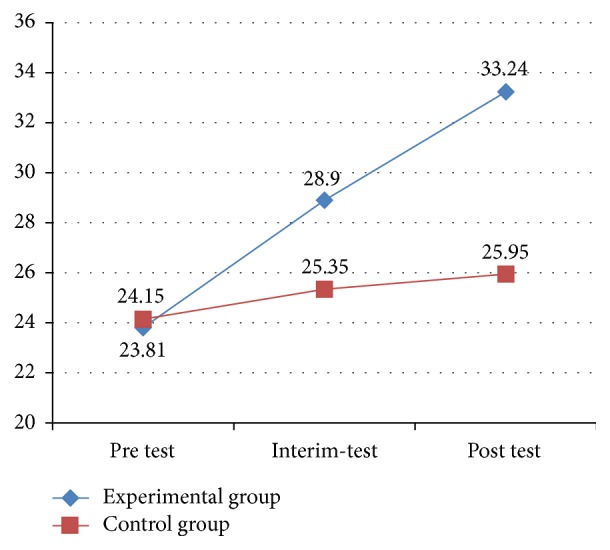
Results of social interaction in ABLLS-R between two groups.

After the 12-week structured physical activity program, an even more significant improvement was found in social interaction scores (*p* < 0.005) for the experimental group compared with the control group, which had a statistically significant effect. These results indicated the structured physical activity program had a positive influence on social interaction in the children with ASD and improved their overall social interaction scores in ABLLS-R (see Figure 3).

Figure 3 displays the improvement trends in social interaction for the experimental group and control group, respectively. The figure shows that the experimental group exhibited a more noticeable improvement over time, with significant differences in social interaction from pretest to interim-test, and then to posttest. By comparison, no significant differences were found in social interaction among the three testing periods for the control group.

#### 3.2.2. Within-Group Comparison of Social Interaction for Two Groups across Three Tests

As shown in Table 8, two-way repeated measure ANOVA was conducted to examine whether a significant difference existed at three time points for the within-group comparison. The results revealed significant differences among T1, T2, and T3 for the experimental group (all *p* < 0.005). Furthermore, the overall social interaction scores observed at T3 were significantly higher than those at T1. By comparison, the control group also exhibited some changes; however, there were no statistically significant differences in the overall social interaction scores across the three time points (all *p* > 0.005). These results showed that the structured physical activity program had a positive influence on the social interaction in the experimental group and improved their overall social interaction scores in ABLLS-R.

#### 3.2.3. Evaluation of Effective Results on Social Interaction

In order to interpret the significant differences on social interaction (*p* < 0.005) between experimental and control group, both effect results of between/within-subject groups had been evaluated through the Partial Eta Squared. The results were showed in the tables (Tables 9 and 10).

Table 9 lists the results of the within-subjects effects. As shown in the table, the results of the within-subjects tests showed that time generated a statistically significant difference on the measured indicator over time (*p* < 0.005). In addition, the interaction effect between the testing time and group had a statistically significant effect (*p* < 0.005), indicating that time influenced the participants differently according to the group they were in. In other words, time influenced the two groups differently.

As displayed in Table 10, the results regarding the between-subjects effects revealed that the group factor generated a significant influence on social interaction for both groups (*p* < 0.005).

### 3.3. The Effects of Structured Physical Activity Program on Communication

#### 3.3.1. Effects of Intervention on Communication across Test Intervals

*(1) Between-Group Comparison of Overall Communication across Three Time Points*. In relation to the scores of communication in SSIS between the experimental group and control group, two-way repeated measure ANOVA was conducted to examine whether the physical activity program resulted in an increase in communication between the experimental and control groups.

As shown in Table 11, after the 12-week physical activity program, a significant improvement was found in communication for experimental group, compared with control group. In addition, there was a significant interaction between group and time (pretest, interim-test, and posttest) of assessment. These results indicated physical activity program had a positive influence on communication for the children with ASD and improved their overall scores of communication in SSIS.

**Figure 4:**
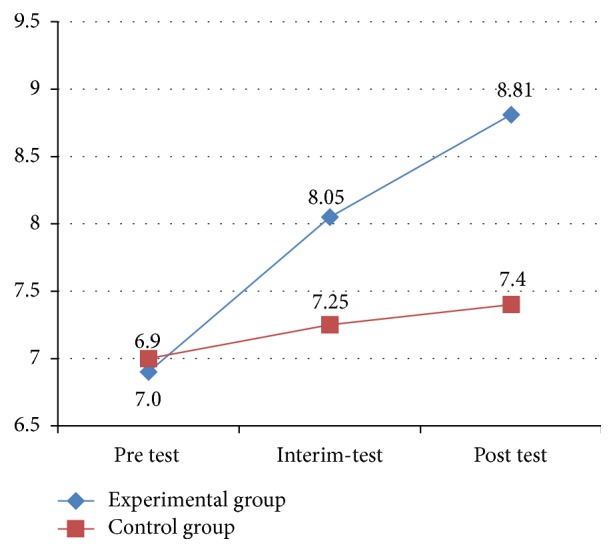
Results of communication in SSIS between two groups.

Figure 4 displays the improvement trends in communication for experimental group and control group, respectively. The figure shows that the experimental group exhibited a more noticeable improvement over time, with significant differences in social skills from the pretest to the interim-test, and then to the posttest. By comparison, no significant differences were found in communication among the three testing periods for the control group.

*(2) Within-Group Comparison of Overall Communication across Three Time Points*. The overall communication scores for the experimental and control groups were measured across three time intervals including baseline (T1), interim-test (T2), and posttest (T3).

As reported in Table 12, two-way repeated measure ANOVA was conducted to examine whether a significant difference existed at the three time points for the within-group comparison. The results revealed significant differences observed from T1 to T3 (*p* < 0.005). By comparison, the control group exhibited no statistically significant differences in the overall communication scores across three time points (all *p* > 0.005). These results indicated that physical activity program had a positive influence on communication in experimental group and improved their overall communication scores in SSIS.

Two-way repeated measures ANOVA was conducted to compare 7 items in communication on three different times (one week before the intervention, the eighth week of the intervention, and after the 12-week intervention). Among the 7 items of communication a statistically significant effect was found in “Says thank you” and “Makes eye contact when talking” (*p* < 0.005). For all comparisons, the significance of the mean difference was set at the .005 level. Conversely, the control group did not show significant changes from pre- to interim-, to posttest on the same items.

*(3) Evaluation of Effective Results on Communication*. In order to interpret the significant differences on communications (*p* < 0.005) between experimental and control group, both effect results of between/within-subject groups had been evaluated through the Partial Eta Squared. The results were showed in the tables (Tables 13 and 14).

As displayed in Table 13, the results of the between-subjects effects showed that the group factor did not generate a significant influence on communication for the two groups (*p* > 0.005)

As reported in Table 14, the results regarding within-subject tests revealed that the time factor generated a statistically significant difference (*p* < 0.005) on the measured indicator (social interaction) over the time.

In addition, the interaction between testing time (pretest, interim-test, and posttest) and group had a statistically significant effect (*p* < 0.005), indicating that time influenced the participants differently according to the group they were in. In other words, the measurement time influenced the two groups differently.

### 3.4. Parents' and Volunteers' Perceptions on the Effects of Structured Physical Activity Program on Social Interaction and Communication

The PSSIG results were used to answer the third research question. 13 parents (8 mothers and 5 fathers) with a mean age of 34.7 years old and 10 volunteers (4 male and 6 female) with the mean age of the parents being 23.2 years old participated in the interview and survey. They all had experiences working with children with disabilities.

#### 3.4.1. Parents' and Volunteers' Perceptions on Overall Effects of Structured Physical Activity Program

*Parents' Perceptions*. The parents' perceptions of the physical activity program were determined by conducting a semistructured interview, which was composed of five parts and 12 questions. It includes warm-up questions, parents' perceptions, and thoughts on the physical activity program, the effects of the physical activity program on social interaction and communication, the beneficial and influential aspects of the program, and future directions and recommendations for obtaining a more practical guide for achieving further improvements in the future. Overall, the parents believed that the structured physical activity program had a positive effect on children' social interaction and communication skills and that all of the children with ASD benefited considerably from participating in the 12-week physical activity program.

*Volunteers' Perceptions*. The volunteers' perceptions of the physical activity program were determined through an open-ended questionnaire, which was composed of four parts and 10 questions. It includes the respondent's information, participants' perceptions of the physical activity program and their experience with it, the effects of the program on the beneficial aspects of the intervention, future directions, and recommendations for obtaining a more practical guide for achieving further improvements in the future. Compared with the parents, all 10 volunteers indicated that they enjoyed the volunteer work with the participants each week as well as in the physical activity program for 12 weeks; the program enabled them to enjoy building a relationship with the children with ASD while observing their weekly progress.

#### 3.4.2. Parents' and Volunteers' Perceptions on the Effects of Physical Activity Program on Social Interaction

**Figure 5:**
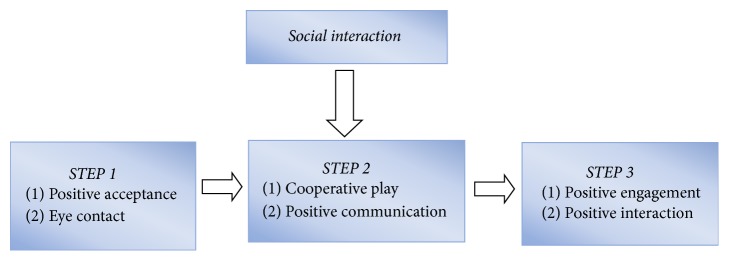
Different developmental steps in social interaction.

After the 12-week intervention, a positive influence was found on social interaction from participating in the physical activity program, according to the perspectives of the parents and participants. Based on the findings of the semistructured interview, three themes were determined, which were actually three steps how the social interactions skills is gradually developed (see Figure 5).

As Figure 5 indicated, the developmental process of social interactions comprises three basic steps: Step 1 includes positive acceptance and eye contact. Step 2 includes cooperative play and positive communication. Step 3 includes positive engagement and positive interactions.

#### 3.4.3. Parents' and Volunteers' Perceptions on the Effects of Structured Physical Activity Program on Communication

Communication skills play a critical role in daily life for everyone, especially for children with communication impairment. This physical activity program purposefully provided many opportunities to raise the level of communication in a natural environment where communication skills could be developed and improved upon, and where the participants were encouraged to learn how to interact with others and how to express themselves, so that they could improve their communication skills. According to the program's requirements, certain purposefully designed interactive activities and teaching tools were used as the key components, and they all had beneficial effects on participant outcomes.

**Figure 6:**
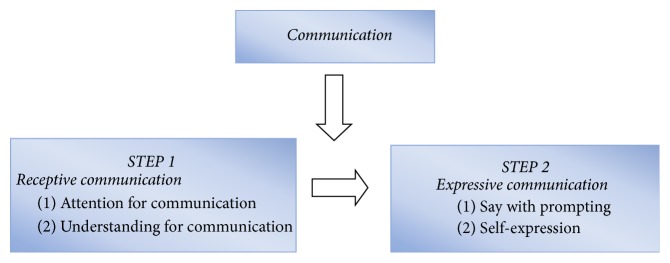
Different developmental steps in communication.

The results of the survey showed that the participants gained different communication benefits from the 12-week physical activity program. The participants exhibited varying degrees of improvement in these two steps of communication development because of individual differences and their original communication level.  Figure 6 displays the two steps of communication development and the subthemes, which involve various steps.

## 4. Discussion

The present study examined the effects of a 12-week structured physical activity program on social interaction and communication for children with ASD. The results of this study provide evidence that structured physical activity is effective on certain autistic characteristics of children with ASD. Although the current study has not identified any single approach that is superior to all other methods, researchers have found a number of effective strategies that can yield obvious improvements on social and communication skills in children with ASD.

### 4.1. The Effects of Structured Physical Activity Program on Social Interaction

According to Reid & Collier [26], there was a positive relationship between physical activity and social interaction, and physical activity and sports participation were beneficial in enhancing interpersonal relationship and increasing frequency of social interaction. The results of SSIS from present study indicated that physical activity program intervention had a positive influence on the social skills for the children with ASD and improved their overall scores of social skills in SSIS. Among all 7 subdomains of SSIS, the results revealed statistically significant differences in the subdomains of communication, cooperation, and self-control.

Similar to the SSIS, the ABLLS-R results showed that there was a positive influence on the social interaction for the children with ASD through participating in the 12-week physical activity program. The results of the current study supported and confirmed the finding of previous studies which demonstrated physical activity can improve social development in children with ASD and contributed to the improvement of social interaction. The findings of the current study are also similar to the findings of three previous studies [19, 27, 28] which all showed that physical activities positively affect social interaction in children with ASD.

One possible explanation for the effectiveness of the physical activity program on social interaction was that (1) physical activity has been regarded as the natural settings for promoting positive social interactions for children with ASD [13, 18]. It could provide a natural environment for building relationships between participants, increase the opportunities for interaction, and offer a good approach to engaging in cooperative play or partnering for teamwork, and presenting more opportunities to communicate with others, which are all beneficial for social interaction. (2) The program was designed based on the guidelines, recommendations on physical activity, some previous successful researches, the development characteristics, and the special needs for children with ASD. In addition, throughout the program, social interaction became the target of all the regular activities, and it purposely integrated certain useful and meaningful elements into the program to present more opportunities for social interaction. The structured program specialized in social interaction and communication, including four coherent content parts; specifically, when the participants finished the entire session as well as after constant practice, the participants may have already increased the frequency of social interactions and improved their abilities for social interaction. For example, encouraging the participants to seek help from each other, facilitating sharing exchanges during group games and activities, addressing some skills and manners, lining up for a turn, and even occurring in some noninstructional socialization cases. All the activities in the program were implemented with the goal of making the social interaction mutually reinforcing to both the children with ASD and their teachers and volunteers.

In addition, the teachers and volunteers conducted the same activity, same exercises, and same skills, which also provided opportunities for participants from watching the positive social interaction of others in order to follow and practice. During the intervention, it provided small group at a 1 : 5 teacher-to-children ratio and 1 : 2 volunteer-to-children ratio and repeated the same contents over and over. These designs may produce positive feedback provided by each teacher and volunteer and then conduct more individualized instruction and practice. Every five participants were paired up with the same teacher and same volunteers for each session, and each session followed the same contents and schedule in order to provide the participants the sense of consistency for completing the program more effectively. Therefore, the potential positive influences of the program were maximized.

Another possibility accounting for the improvement of social interaction through the intervention was that the program utilized the structured teaching of TEACCH model, one of the most validated treatment programs used with individuals with autism (e.g., physical structure, schedule visual card, and work systems) (Panerai, Ferrante & Zingale, 2002). For example, each participant had his or her own schedule visual card, which showed the activities that had to be conducted. Every time the participants entered the gym, they had to consult their individual schedule card and perform the activities as shown on the schedule and use the physical boundaries to foster independent behavior for participants and increase the level of participants' emotional security. The design of boundaries allowed for maximum time on task for participants through decreasing external stimuli that might have distracted the participants.

### 4.2. The Effects of Structured Physical Activity Program on Communication

Communication skill plays a critical role in daily life for children with ASD. During this physical activity program, it purposely provided numerous opportunities for enhancing the level of communication in a natural and specific environment in which communication skills could develop further. Moreover, the participants were encouraged to learn how to interact with others and how to express themselves, so that they could improve the communication skills for them.

First, the SSIS was used to answer the second question. After the 12-week structured physical activity program, significant differences were observed in the experimental group, and it indicated that structured physical activity program intervention had a positive influence on communication for the children with ASD and improved their overall scores of communication in SSIS. Among all the 7 items of communication, the results revealed statistically significant differences in “says thank you” and “makes eye contact when talking.”

Second, two qualitative instruments, PSSIG and VOEQ, were also used to assess the effects of structured physical activity program on communication from the perspectives of parents and volunteers in order to supply and furnish the quantitative findings. The qualitative results were similar to the quantitative results. Overall, after the 12-week physical activity program parents and volunteers all noted a positive improvement in the children's overall communication. According to the reports of the parents and participants, an obvious improvement primarily appears in aspects of understanding in communication and saying with prompting.

The results of this study supported and confirmed that findings of a previous study that reported that physical activity improves communication skills [19]. And our findings were also similar to those reported in two past studies [27, 28] which found that physical activities positively affected social skills, communication, and motor skills in children with ASD.

In this study, the physical activity program, which was a structured program with purposeful activities, a targeted design, and teaching tools (e.g., visual cue cards, activity schedule, PowerPoint, and a large screen), was utilized for the beneficial communication outcomes. Moreover, we incorporated the structured teaching of the TEACCH model as a beneficial treatment strategy for facilitating language development, so that we could create a comfortable environment to positively influence the children's communication skills. These findings also supported the effectiveness of Schopler's (1966) TEACCH model as a beneficial treatment strategy for facilitating language development [29].

One possible explanation for the effectiveness of the physical activity program in communication was that during the whole program all the regular activities were highly targeted on problem of communication, and it purposely integrated certain useful and meaningful elements into the program in order to create more opportunities to enhance the level of communication. Prompting and encouragement were used throughout the physical activity program to encourage communication among the participants. During the various sessions, both the teachers and volunteers posed many basic questions to increase opportunities for communication, and to encourage the participants to speak (e.g., asking questions concerning their names and their favorite toys, encouraging everyone to sing songs together, and repeating their name in each part of the session).

Another possibility accounting for the improvement of communication skills through the intervention was the design of small group. There were 1 : 5 teacher-to-children ratio and 1 : 2 volunteer-to-children ratio, which may produce more effective and positive results, and also clear feedback was provided by each teacher and volunteer. The teachers and volunteers were able to follow the progression of each participant at his or her own pace and teach more effectively associated with the participant's disability and the rate of progress. During the whole program, every five participants were paired up with the same teacher and same volunteers for each session, and each session followed the same contents and schedule in order to provide the participants the sense of consistency for completing the program more effectively. Therefore, the potential positive influences of the program were maximized.

In addition, the program had the reward system to encourage the partisans to be active and be willing to participate in the activities of the program. For example, when children finished the exercise, the teachers gave them reward, and then they may say thank you and bye-bye or have an eye contact, which were the communication improvement. All the activities in the program were implemented with the goal of making the communication mutually reinforcing to both the children with ASD and their teachers and volunteers. When the participants finished the whole session and after constant practice, the participants may have already increased the frequency of communication and improved the ability of communication.

### 4.3. The Beneficial and Influential Elements of the Physical Activity Program

To obtain valuable results, the intervention setting was considered to be of utmost importance. Two qualitative instruments were used to answer the fourth question and identify the beneficial and influential factors of the structured physical activity program. Based on the responses of the parents and volunteers, these factors included the following: (a) physical activity; (b) purposeful design; (c) a structured program; (d) teaching tools; (e) low teacher-student and volunteer-student ratio; and (f) a comfortable environment.

A number of these beneficial factors have been supported in previous findings and have been found to positively affect the physical activity program [30, 31]. The findings of this study are also similar and support the notion that certain components of the program influenced the effectiveness of social interactions and communication for children with autism [30, 31]. Moreover, it revealed that multiple strategies are influential in the success of a physical activity program for children with ASD. Based on the responses from volunteers and parents, each element was regarded as having positive influence on the children with ASD, but actually it was the combination of these elements that was believed to result in the noticeable improvement in social interaction and communication.

In order to interpret the significant differences on social interaction and communication (*p* < 0.005) between experimental and control group, both effect results of between/within-subject groups had been evaluated through the Partial Eta Squared. The results had supported the significant improvement that occurred in the experimental group. Overall, the combination of all the beneficial and influential components has resulted in an effective physical activity program in which the children can participate, play, and develop their skills for social interaction and communication.

## 5. Conclusions

This study examined the effects of a 12-week structured physical activity program on social interaction and communication in children with ASD. After the 12-week physical activity program, the results showed an overall improvement on social interaction and communication skills for the experimental group compared with the control group. (1) Regarding social interaction, the study had found significant improvements in social interaction, cooperation, and self-control subdomains. However, the effects of physical activity program were minor in terms of cooperation, responsibility, empathy, and engagement. The 12-week intervention might not have been sufficient in length to generate many obvious changes in every aspects of social interaction. The parent's interviews and volunteers' questionnaire responses indicated significant changes in the overall level of social interaction by the end of the 12-week physical activity program. Certain social skills (e.g., eye contact, group participation, and relationship-building with teachers and participants) were found improved. (2) Regarding communication, the results revealed an overall improvement for the experimental group compared with the control group; the improvements were observed in the frequency of reacting peers, teachers, and volunteers' greeting by “says thank you” and “makes eye contact when talking.” The results reflected the perspectives expressed by the parents and volunteers. (3) The 12-week physical activity program was found to be effective on social interaction and communication for children with ASD according to the results across three test intervals and feedback from of teachers, parents, and participants. It indicated that the structured physical activity program with a purposeful design and naturally integrated social interaction/communication elements into the regular physical activity program is effective in promoting positive improvements in certain impairments for children with ASD.

In summary, this study was the first step towards gaining a better understanding of physical activity program intervention on children with ASD, furnishing useful materials, information, and practice to facilitate, define, and measure the effects of physical activity program for future researches. It also provided a broad picture of the insights in development of more comprehensive and inclusive programs specialized for children with ASD, and fulfilled the gaps of research in physical activity intervention on children with ASD in China.

## Figures and Tables

**Table 1:** The demographic data of the participants.

Variables	Experimental group (*N* = 21)	Control group (*N* = 20)	Results
Age, mean (SD)	6.14 (0.96)	6.1 (0.98)	*p* = 0.889
Gender			
Boys	14 (66.67%)	15 (75.00%)	*p* = 0.558
Girls	7 (33.34%)	5 (25.00%)	
Diagnosis			
Classic autism	17 (80.95%)	16 (95.00%)	*p* = 0.563
Asperser's syndrome	3 (14.29%)	4 (5.00%)	
PDD	1 (4.76%)	0 (0%)	

**Table 2:** Physical activity program protocol.

Part	Time (min)	Content	Objective
(1) Get-ready and warm-up activity	10	Get the visual schedule cards; Prepare the equipment; Jogging on the playground	Social interaction and communication skills

(2) One-to-five small group instruction	15	Learn the skills of playing ball (tap, throw, catch, pass the ball) Follow the teachers and practice Play the ball exercises and games	Social interaction and communication skills

(3) Whole group exercise	20	Group ball exercise; Cooperative ball games	Social interaction and communication skills

(4) Cool-down and reward activity	15	High five to teachers, volunteers and partners; Sing good bye songs together; Get the reward	Feedback, social interaction and communication skills

**Table 3:** Data collection schedule.

Instruments	Participants	Before the intervention (pretest)	Physical activity program	Week 7 of intervention (interim-test)	After the intervention (posttest)
ABLLS-R	Teachers	●		●	●
SSIS	Teachers	●		●	●
PSSIG	Parents				●
VOEQ	Volunteers				●

**Table 4:** *Between-group comparison of overall social skills across three* intervals.

Group	Pretest	Interim-test	Posttest
Control group	44.05 ± 4.66	45.20 ± 5.16	45.15 ± 5.03
Experimental group	43.95 ± 6.96	48.52 ± 5.62	51.57 ± 5.47^*∗∗*^
Results	*p* = 0.958	*p* = 0.049	*p* = 0.000

*Note*. *∗∗* represented *p* < 0.005, and results are presented as means ± SD.

**Table 5:** The Subdomains of social skills in SSIS across time by experimental group and control group and results on comparison.

Index	Control Group (*n* = 20)			Experimental Group (*n* = 21)		
	Pre	Interim	Post	Pre	Interim	Post
Communication	7.00 ± 1.56	7.25 ± 1.59	7.40 ± 1.47	6.90 ± 1.84	8.05 ± 1.43	8.81 ± 1.60^*∗∗*^
Cooperation	7.55 ± 1.43	7.50 ± 1.28	7.65 ± 1.31	7.57 ± 1.54	8.19 ± 1.57	8.48 ± 1.63^*∗∗*^
Assertion	4.50 ± 1.10	4.90 ± 1.33	5.00 ± 1.30	4.43 ± 1.40	4.95 ± 1.56	4.57 ± 1.50
Responsibility	6.05 ± 1.15	6.20 ± 1.20	6.04 ± 1.14	6.00 ± 1.26	6.57 ± 1.25	6.80 ± 1.25
Empathy	5.55 ± 1.46	5.60 ± 1.15	5.60 ± 0.99	5.57 ± 1.36	5.90 ± 1.26	6.24 ± 1.30
Engagement	6.55 ± 1.19	6.95 ± 1.19	7.20 ± 1.11	6.33 ± 1.29	7.43 ± 1.16	7.76 ± 1.48
Self-control	6.85 ± 1.60	6.75 ± 1.45	6.70 ± 1.75	7.14 ± 1.77	7.43 ± 1.78	7.90 ± 1.55^*∗∗*^

*Note*. *∗∗* represented *p* < 0.005 and results are presented as means ± SD.

**Table 6:** Within-group comparison of overall social skills for two groups across three time intervals.

Group	Time	M ± SD	T1-T2		T1–T3		T2-T3	
			Mean		Mean		Mean	
			Difference	Sig.	Difference	Sig.	Difference	Sig.
Experimental group	T1	43.95 ± 6.96	−4.574	.020	−7.621	.000	−3.048	.111
	T2	48.52 ± 5.62						
	T3	51.57 ± 5.47						

Control group	T1	44.05 ± 4.66	−1.150	.474	−1.200	.455	−.050	.975
	T2	45.20 ± 5.16						
	T3	45.15 ± 5.03						

**Table 7:** Between-group comparison of overall social interaction in ABLLS-R across tests.

Group	Pretest	Interim-test	Posttest
Control group	24.15 ± 3.34	25.35 ± 5.78	25.95 ± 2.87
Experimental group	23.81 ± 3.43	28.90 ± 3.18^*∗∗*^	33.24 ± 3.79^*∗∗*^
Results	*p* = 0.749	*p* = 0.001	*p* = 0.000

*Note*. *∗∗* represented *p* < 0.005, and results are presented as means ± SD.

**Table 8:** Within-group comparison of social interaction for two groups at three time points.

Group	Time	M ± SD	T1-T2		T1–T3		T2-T3	
			Mean		Mean		Mean	
			Difference	Sig.	Difference	Sig.	Difference	Sig.
Experimental group	T1	23.81 ± 3.43	−5.095	.000	−9.429	.000	−4.333	.000
	T2	28.90 ± 3.18						
	T3	33.24 ± 3.79						

Control group	T1	24.15 ± 3.34	−1.200	.242	−1.800	.081	−.600	.556
	T2	25.35 ± 5.78						
	T3	25.95 ± 2.87						

**Table 9:** Within-subject effects results.

Source	Type III sum of squares	df	Mean square	*F*	Sig.	Partial Eta Squared
Time	405.169	2	202.584	88.715	.000	.695
Time *∗* group	214.730	2	107.365	47.017	.000	.547
Error (time)	178.116	78	2.284			

**Table 10:** Between-subjects effects results.

Source	Type III sum of squares	df	Mean square	*F*	Sig.	Partial Eta Squared
Intercept	88953.707	1	88953.707	3266.72	.000	.988
Group	376.634	1	376.634	13.832	.001	.262
Error	1061.967	39	27.230			

**Table 11:** Between-group comparison of overall communication across three tests.

Group	Pretest	Interim-test	Posttest
Control group	7.00 ± 1.56	7.25 ± 1.59	7.40 ± 1.47
Experimental group	6.90 ± 1.84	8.05 ± 1.43	8.81 ± 1.60^*∗∗*^
Results	*p* = 0.859	*p* = 0.098	*p* = 0.004

*Note*. *∗∗* represented *p* < 0.005 and results are presented as means ± SD.

**Table 12:** Within-group comparison of communication for two groups across three tests.

Group	Time	M ± SD	T1-T2		T1–T3		T2-T3	
			Mean		Mean		Mean	
			Difference	Sig.	Difference	Sig.	Difference	Sig.
Experimental group	T1	6.90 ± 1.84	−.707	.053	−1.171	.002	−.463	.202
	T2	8.05 ± 1.43						
	T3	8.81 ± 1.60						

Control group	T1	7.00 ± 1.56	−.250	.609	−.400	.414	−.150	.202
	T2	7.25 ± 1.59						
	T3	7.40 ± 1.47						

**Table 13:** Between-subjects effects results.

Source	Type III sum of squares	df	Mean square	*F*	Sig.	Partial Eta Squared
Intercept	7041.799	1	7041.799	1055.783	.000	.964
Group	15.230	1	15.230	2.283	.139	.055
Error	260.120	39	6.670			

**Table 14:** Within-subject effects results.

Source	Type III sum of squares	df	Mean square	*F*	Sig.	Partial Eta Squared
Time	27.602	2	13.801	31.266	.000	.445
Time *∗* group	11.732	2	5.866	13.290	.000	.254
Error (time)	34.430	78	.441			
